# A Global Biomedical R&D Fund and Mechanism for Innovations of Public Health Importance

**DOI:** 10.1371/journal.pmed.1001831

**Published:** 2015-05-11

**Authors:** Manica Balasegaram, Christian Bréchot, Jeremy Farrar, David Heymann, Nirmal Ganguly, Martin Khor, Yves Lévy, Precious Matsoso, Ren Minghui, Bernard Pécoul, Liu Peilong, Marcel Tanner, John-Arne Røttingen

**Affiliations:** 1 Access Campaign, Médecins Sans Frontières, Geneva, Switzerland; 2 Institut Pasteur, Paris, France; 3 Wellcome Trust, London, United Kingdom; 4 Centre on Global Health Security, Chatham House, London, United Kingdom; 5 Jawaharlal Institute of Postgraduate Medical Education & Research, Puducherry, India; 6 South Centre, Geneva, Switzerland; 7 INSERM, Paris, France; 8 Department of Health, Pretoria, South Africa; 9 Department of International Cooperation, China National Health and Family Planning Commission, Beijing, People’s Republic of China; 10 Drugs for Neglected Diseases initiative, Geneva, Switzerland; 11 Department of Global Health, School of Public Health, Peking University, Peking, China; 12 Swiss Tropical and Public Health Institute, Basel, Switzerland; 13 Norwegian Institute of Public Health, Norway, University of Oslo, Olso, Norway; 14 Harvard T. H. Chan School of Public Health, Boston, Massachusetts, United States of America

## Abstract

Bernard Pécoul and colleagues call for the establishment of a global biomedical R&D fund as a key priority of the G7 summit in June 2015.

**Summary Points**

- Anti-microbial resistance, emerging infectious diseases, and neglected diseases are all important public health concerns and priorities with serious market failures, deficits, and identified needs in biomedical innovation.
- It is important to reconcile, rather than fragment, the needs of these three priority areas by considering an umbrella framework for specifically financing and coordinating research and development (R&D) that delivers innovation while securing patient access.
- A sizeable, sustainably financed global R&D fund and mechanism that promotes coordination, collaboration, and utilization of new and innovative incentives should be set up to cover all three priority areas.

Over the last few years, there have been significant challenges in and increased concerns around anti-microbial resistance (AMR), emerging infectious diseases, and neglected diseases (NDs). Both AMR and emerging infectious diseases, specifically Ebola, have recently been elevated to the level of public health concerns affecting global security. All these areas have identifiable prevention and management strategies that can and should be scaled up. However, an additional consistent thread that runs throughout all these global health concerns is the deficit in innovation of new tools in relation to the identified needs. Therapeutics and vaccines for Ebola remain experimental and treatments for many neglected diseases remain archaic, while the current antibiotic pipeline is drying out and will most likely not meet current and future challenges, even if efforts to rationally use these medicines are stepped up.

The reasons behind these failures of the research and development (R&D) system are multi-faceted and have been comprehensively described before and widely acknowledged by the World Health Organization (WHO) and its member states [1]. The consultative expert working group (CEWG) on R&D Financing and Coordination at WHO reviewed more than 100 proposed ideas and suggested the need for alternative models to fund and incentivize R&D, supported by a global normative R&D framework that would deliver both innovation and access, underpinned by certain key principles and a pooled R&D fund [2]. Some aspects of these models have already been utilized for NDs and are being explored for antibiotics. The CEWG recommendation for the establishment of an international treaty including mandatory financial contributions from WHO member states has not achieved widespread support. However, WHO member states have recently indicated support for establishing a fund with a voluntary financing model that would finance biomedical R&D based on the principles formulated by the CEWG, namely: de-linkage of the delivery price from R&D costs, the use of open knowledge innovation, and licensing for access [3,4]. Such principles would allow for lower cost and more efficient R&D centered on the needs and resources of the people who need them most. Of note, while large, international, multi-lateral funds exist for global health delivery (e.g., UNITAID; The Global Fund to Fight AIDS, Tuberculosis and Malaria; the Gavi Vaccine Alliance), there is no significant large pooled funding mechanism for infectious disease R&D that works as a multi- or poly-lateral (i.e., with both state and non-state actors) mechanism.

The idea of a global financing mechanism for innovation has been discussed for global health priorities in general, NDs, antibiotics, and more recently, Ebola [5–7], but in separate discussions. All of these are clearly public health priorities, and some may constitute public health emergencies of international concern (as defined in the International Health Regulations). They provide limited, unpredictable, and therefore unattractive commercial markets. Many are also priorities for all countries across the low-, middle-, and high-income groupings, and for all these diseases the medical tools needed to address them should be considered as global public goods.

Before jumping to create multiple new mechanisms, it would make sense to consider reconciling the needs of all these areas by considering an umbrella framework for specifically funding and coordinating R&D that not only emphasizes innovation but also secures access. During the last WHO Executive Board, member states stressed the importance of creating such a framework, citing the Ebola crisis and AMR to justify its urgent need. From our perspective, such a framework could provide longer term vision, clear priorities, and increased capacity for risk-taking, beyond what is feasible given the political constraints of individual national or regional sources of funding. It could address global needs and gaps based on target product profiles that can serve a range of contexts, supporting and incentivizing a wide range of existing and new research entities, public and private, across low-, middle-, and high-income economies. It could also combine orthodox (e.g., grants) and less traditional incentives (e.g., prizes, bonds), while promoting the sharing and pooling of knowledge, data, and technologies. The use of such strategies should be strongly coupled and assist with delivering equitable access to the fruits of innovation.

However, there are some clear prerequisites required to enable a functional R&D framework. First, we need a specific R&D financing mechanism that, in turn, would need to be linked closely with one or more entities that have the ability to monitor research flows and outputs [8]. The mechanism would need to be able to set global priorities according to agreed-upon needs and to play a key role in formulating a global strategy, in collaboration with other actors and funders. Such normative functions mean it would require a strong link with an inter-governmental agency like the WHO. Further evaluation is required before it can be considered whether the mechanism needs to sit inside or alongside the United Nations system, and this may be dependent upon parallel issues such as WHO reform and potential post-Ebola reforms. The mechanism would need to be essentially publicly financed and owned and overseen by governments, but private and philanthropic actors and civil society should be involved as stakeholders. Good governance to ensure oversight and transparency and an effective, professional, and suitably competent secretariat will be essential. Moreover, the mechanism must strive to be lean and public-health–oriented, with clear performance measures, in order to more rapidly deliver needed innovations to patients. The crisis-response R&D processes elicited by the Ebola outbreak demonstrate that such a mechanism could also be useful by allowing a more proactive and expeditious approach to address emerging public health needs.

Both the CEWG and the Lancet Commission on Investing in Health have called for a doubling of global health R&D funding to US$6 billion annually for infections of poverty [2,6]. An additional investment of around US$4 billion would be needed for AMR and emerging infections, totaling US$10 billion. Echoing Jim O’Neill’s (Chair of the UK government’s review on AMR) recent call for a US$2 billion AMR fund, at least US$2–3 billion should be internationally pooled [9]. This is necessary to secure sufficient strength of coordination across funders. However, the fund should not become one large, monopolistic, and monolithic operator. It should, rather, be in tandem with other large public and private funders, and promote alignment, collaboration, and synergy to build a broad investment strategy and portfolio, thereby hedging for failures, which inevitably are part of the process of biomedical innovation. New and additional sources of financing would be required, notably from emerging economies, as well as innovative financing mechanisms.

The events of 2014 have shown that crisis management is more risky and costly, and less effective, than a prepared health system. This also holds true for the innovation needs, and demonstrates the need for a preparedness R&D mechanism that can quickly deliver innovative responses to emerging health threats. With a stronger R&D mechanism in place, innovative vaccines and therapeutics could have been ready for testing earlier on in the Ebola outbreak in West Africa. The need for change must be a call to action to mobilize countries to take collective action. We believe that such a move that transcends state and corporate interests would result in a win-win scenario for all—rich and poor populations, and public and private sectors alike.

The devastating loss of human life from the Ebola outbreak of 2014 must not be in vain. It must prompt serious changes to our joint international systems for stimulating innovation and ensuring access to health technologies for those who need them. These issues must be on the agenda for the evaluation panels established in the aftermath of Ebola, at World Health Assembly and UN levels. It must also be a key priority at the G7 summit in June 2015 [10]. We call for one of their recommendations to be the establishment of a global biomedical R&D fund and mechanism for innovations of public health importance.

## Abbreviations

AMR: anti-microbial resistance
CEWG: consultative expert working group
NDs: neglected diseases
R&D: research and development
